# Publisher Correction: Antiviral and clinical activity of bamlanivimab in a randomized trial of non-hospitalized adults with COVID-19

**DOI:** 10.1038/s41467-023-35835-3

**Published:** 2023-01-19

**Authors:** Kara W. Chew, Carlee Moser, Eric S. Daar, David A. Wohl, Jonathan Z. Li, Robert W. Coombs, Justin Ritz, Mark Giganti, Arzhang Cyrus Javan, Yijia Li, Manish C. Choudhary, Rinki Deo, Carlos Malvestutto, Paul Klekotka, Karen Price, Ajay Nirula, William Fischer, Veenu Bala, Ruy M. Ribeiro, Alan S. Perelson, Courtney V. Fletcher, Joseph J. Eron, Judith S. Currier, ACTIV-2/A5401 Study Team, Michael D. Hughes, Davey M. Smith

**Affiliations:** 1 Department of Medicine, David Geffen School of Medicine at University of California, Los Angeles, Los Angeles, CA USA; 2 Harvard T.H. Chan School of Public Health, Boston, MA USA; 3 Lundquist Institute at Harbor-UCLA Medical Center, Torrance, CA USA; 4 Department of Medicine, University of North Carolina at Chapel Hill School of Medicine, Chapel Hill, NC USA; 5 Department of Medicine, Brigham and Women’s Hospital, Harvard Medical School, Boston, MA USA; 6 Department of Laboratory Medicine and Pathology, University of Washington, Seattle, WA USA; 7 Department of Medicine, University of Washington, Seattle, WA USA; 8 National Institutes of Health, Bethesda, MD USA; 9 Ohio State University Wexner Medical Center, Columbus, OH USA; 10 Eli Lilly and Company, San Diego, CA USA; 11 UNMC Center for Drug Discovery, University of Nebraska Medical Center, Omaha, NE USA; 12 Theoretical Biology and Biophysics Group, Los Alamos National Laboratory, Los Alamos, NM USA; 13 Department of Medicine, University of California, San Diego, La Jolla, CA USA; 14 Present Address: Department of Medicine, University of Pittsburgh, Pittsburgh, PA USA; 15 Present Address: Clinical Pharmacology & Pharmacometrics, Jounce Therapeutics, Cambridge, MA USA

**Keywords:** Viral infection, Medical research, SARS-CoV-2

Correction to: *Nature Communications* 10.1038/s41467-022-32551-2, published online 22 August 2022

The original version of this article contained an error in the Abstract, which incorrectly omitted from the end the following: ‘Trial Registration: ClinicalTrials.gov Identifier: NCT04518410’. This has been corrected in both the PDF and HTML versions of the Article.

